# Evolution of the monitoring and evaluation strategies to support the World Health Organization's Global Programme to Eliminate Lymphatic Filariasis

**DOI:** 10.1093/inthealth/ihaa084

**Published:** 2020-12-22

**Authors:** Patrick J Lammie, Katherine M Gass, Jonathan King, Michael S Deming, David G Addiss, Gautam Biswas, Eric A Ottesen, Ralph Henderson

**Affiliations:** Division of Parasitic Diseases, Centers for Disease Control and Prevention, 1600 Clifton Rd., Atlanta, GA 30333, USA; NTD Support Center, Task Force for Global Health, 325 Swanton Way, Decatur, GA 30030, USA; NTD Department, World Health Organization, 20 Avenue Appia, 1211 Geneva 27, Switzerland; Division of Parasitic Diseases, Centers for Disease Control and Prevention, 1600 Clifton Rd., Atlanta, GA 30333, USA; Division of Parasitic Diseases, Centers for Disease Control and Prevention, 1600 Clifton Rd., Atlanta, GA 30333, USA; NTD Department, World Health Organization, 20 Avenue Appia, 1211 Geneva 27, Switzerland; NTD Support Center, Task Force for Global Health, 325 Swanton Way, Decatur, GA 30030, USA; NTD Support Center, Task Force for Global Health, 325 Swanton Way, Decatur, GA 30030, USA

**Keywords:** disease elimination, lymphatic filariasis, mass drug administration, monitoring and evaluation

## Abstract

The Global Programme to Eliminate Lymphatic Filariasis (GPELF) was established with the ambitious goal of eliminating LF as a public health problem. The remarkable success of the GPELF over the past 2 decades in carrying out its principal strategy of scaling up and scaling down mass drug administration has relied first on the development of a rigorous monitoring and evaluation (M&E) framework and then the willingness of the World Health Organization and its community of partners to modify this framework in response to the practical experiences of national programmes. This flexibility was facilitated by the strong partnership that developed among researchers, LF programme managers and donors willing to support the necessary research agenda. This brief review summarizes the historical evolution of the GPELF M&E strategies and highlights current research needed to achieve the elimination goal.

Development of the Global Programme to Eliminate Lymphatic Filariasis (GPELF) was catalysed by two crucial scientific advances: single-dose antifilarial therapy that led to long-lasting reductions in microfilaremia and a point-of-care antigen test that simplified identification of the infection in endemic communities.^1,2^ Documentation of the effectiveness of these tools, largely through the World Health Organization's (WHO) Special Programme for Research and Training in Tropical Diseases, provided the operational basis for the GPELF's elimination strategy. This strategy begins with baseline mapping followed by treatment of the entire LF-endemic population with at least five annual rounds of mass drug administration (MDA) using albendazole plus either diethylcarbamazine or ivermectin. A stopping decision is then made based on the results of population surveys that are repeated over the course of another 5 y.^2^ If the final survey results remain below the presumed transmission threshold, the programme is recognized as having validated the elimination of LF as a public health problem. Although ‘elimination as a public health problem’ is a concept whose definition still generates debate,^3^ it has been defined operationally for the GPELF as the reduction of markers of infection (microfilaremia or antigenemia) in endemic areas to levels considered not sufficient to support ongoing transmission.

At the advent of the GPELF in 2000, there was already a wealth of programmatic observation from China, Brazil, Fiji and elsewhere that LF could be eliminated in areas under intensive treatment.^1^ Grounded in these earlier experiences that had proven the feasibility of elimination, the newly identified diagnostic and intervention tools could now serve as the engine for the GPELF, while the development of a rigorous monitoring and evaluation (M&E) framework provided the roadmap. Robust M&E strategies are the cornerstone of effective disease elimination programmes, providing countries with essential, standardized guidance on where interventions are necessary, how to document their impact and when they can be stopped. Since its inception, the GPELF has been strengthened by the application and adaptation of M&E guidelines that themselves continue to evolve in response to programme experience and the availability of newer tools.^4^ The history of the GPELF can be viewed through the lens of M&E, recognizing distinct periods represented by different M&E guidelines necessary to accommodate progressively changing programme challenges and needs. Indeed, although the outline of the framework (Table 1) is essentially unchanged from the beginning of the GPELF, its details have been refined and become much more evidence-based.

**Table 1.** Key steps in the evolution of M&E for the GPELF

	Programme stage				
Year of WHO guidance	Mapping	MDA delivery	MDA impact	MDA stopping	Surveillance
1999^9^	Antigen testing of 250 children using lot quality assurance methodology	Coverage assessment—unspecified target and method	Sentinel and spot-check sites	Antigen survey of 3000 school children	Repeat stopping survey
2005^10^	–	Coverage definitions	–	Antigen surveys of 2- to 4-year-old children	–
				Microfilaria surveys in 5–10 sentinel and spot-check sites	
2011^13^	–	Coverage target defined	–	TAS	Repeat TAS
Post-2011^17,18,20^	Confirmatory mapping survey	Standardized coverage survey	–	–	–
		Supervisor's coverage tool			
Under active research	–	Systematic non-compliance	Transmission hotspots	Adaptation of TAS design	Post-validation surveillance

## Launching the GPELF

Initial recommendations for the GPELF's M&E guidelines were developed in 1999 through a series of WHO meetings to provide epidemiologic guidance for nascent LF elimination programmes.^5,6^ At this time, large-scale programme experience was limited to a small number of very active control programmes^1^ and to the very successful LF elimination programme in China.^7^ These early LF experiences, along with the operational lessons from other large-scale public health initiatives (particularly the Expanded Program on Immunization), helped to generate initial practical guidance for expanding national LF programmes. The WHO meeting reports identified approaches to mapping, defined the use of sentinel and spot check sites for programme monitoring and proposed elimination criteria based on testing samples of 3000 school children for filarial antigen, since a foundational assumption of the strategy was that effective large-scale treatment programmes would reduce and prevent infections among children. Many of the specific recommendations included in these original documents, including the use of lot quality assurance sampling,^8^ persist in the WHO guidelines employed today, albeit with certain modifications introduced to make M&E more operationally practical in national programmes (Table 1). Also of note in these original documents was the identification of specific research needs that remain today—for example, the need to develop a sensitive, specific test to detect exposure to filarial infection in order to support surveillance strategies and the need to create banks of parasite material (from both LF and soil-transmitted helminths) to support studies of drug resistance.

## Scaling up

A grant of US$20 million from the Bill & Melinda Gates Foundation (BMGF) in 2000 was catalytic in supporting both the creation of the Global Alliance to Eliminate LF and the initial phase of scaling up the GPELF. Along with the drug donations of albendazole from SmithKline Beecham (now GlaxoSmithKline) and ivermectin from MSD (trade name of Merck & Co. Inc., Kenilworth, NJ, USA), this bolus of funding was arguably the most critical event in shifting LF elimination from concept to practice. Demonstration programmes initially supported by these donations and by additional bilateral funding were established in Ghana, Burkina Faso, Togo, Tanzania, Zanzibar, Nigeria and India, providing opportunities to scale up interventions and test M&E guidance in real-world situations.

This funding also permitted establishment of the LF Support Center in Atlanta, Georgia (USA) where an informal M&E working group was specifically charged by the WHO to support the GPELF in tracking national experiences in following the 2000 Programme Managers Guidelines^9^ and to use this feedback as the basis for developing updated guidance. This revised guidance was published in 2005^10^ and provided important information on programme monitoring, including definitions of coverage and recommendations on the performance of coverage surveys.

Perhaps the most significant change in the 2005 document, however, was the addition of a more complete description of a proposed approach to determine the criteria for stopping the annual MDAs. The steps described were relatively complex and included surveys for microfilaremia in 5–10 sentinel and spot check sites in each ‘implementation unit’ (usually a district), along with antigen surveys both in 2- to 4-year-old children and in 3000 older school-age children. The target threshold for stopping MDA programmes was defined as a prevalence of microfilaremia of <1% in sentinel sites and <0.1% antigenemia in school-age children (school entrants). A repeat survey of 3000 children 5 y after the first stopping MDA survey was proposed as the basis for verification (now termed ‘validation’^3^) that the elimination target was achieved. Implementation of this guidance proved to be a challenge for the national programmes, both in terms of the number of surveys required and the fact that antigen surveys in 2- to 4-year-old children were generally uninformative. As a result, the WHO's NTD Strategic Technical and Advisory Group (STAG) recommended revising the guidelines again.

## Learning how to stop

Challenges with the implementation of the 2005 M&E guidelines were recognized quickly by the LF community and a strong, coordinated response galvanized researchers and programme managers in an effort to define both the optimal diagnostic tool and a practical method for making MDA stopping decisions for the programmes. Fortunately the importance of these research needs for the GPELF was also recognized by the BMGF, whose funding for the GPELF then shifted from the initial catalytic grant to more research-focused objectives. At the same time, expanded bilateral support for national programmes permitted accelerated scale-up and an increased focus on integrating programme delivery.

Because of these new programme demands and the fact that now, more than 5 y into programme delivery, numerous countries and many districts within countries had reached their anticipated stopping points, the need intensified for new, more detailed M&E guidance—especially for the stopping decision. To address these needs, multicountry studies were designed to determine which diagnostic tools were the most reliable and feasible for use in settings where LF prevalence was low following MDA.^11^ These studies identified the rapid filaria antigen test (immunochromatographic test) as the most feasible tool for programme use in *Wuchereria bancrofti*–endemic settings and also reinforced a culture of engagement between the research and programme implementation communities—an engagement that has greatly benefitted the GPELF over the years.

In response to the challenges posed by the 2005 guidelines, the transmission assessment survey (TAS) was subsequently designed to represent a standardized decision-making tool for stopping MDA that was practical to use by country programmes. Still based on the concept that successful elimination of LF could be inferred by demonstrating the absence of infection in children 6–8 y of age, the TAS defined sampling strategies and critical prevalence thresholds for making the stopping decision. The prevalence thresholds for markers of infection that were used to indicate if a TAS passes (<1% antigenemia where *Aedes* is the main vector, otherwise <2%) were initially chosen based on prior experiences of earlier elimination efforts in China and elsewhere. Multicountry studies documented the feasibility of the TAS design in the field^12^ and set the stage for its adoption by the WHO, which released its new round of M&E guidance in 2011.^13^ This guidance required that each ‘evaluation unit’ have three ‘passing’ surveys (i.e. prevalence below the target threshold) repeated at 2-y intervals after at least 5 y of annual MDA. Eligibility for the initial TAS was based on achieving at least 65% coverage for each round of MDA and on an indication that infection prevalence in sentinel and spot-check sites was below the target thresholds (1% for microfilaremia or 2% for antigenemia). The requirement for three TASs introduced a surveillance component into programme M&E and established an operational definition for ‘elimination of LF as a public health problem’, setting the stage for the WHO's ‘validation’ process as a formal acknowledgement of a country's successful completion of programme implementation, scale-up, stopping and post-stopping follow-up. The release of a revised M&E manual in 2011 and the development of training materials to support the introduction and use of the TAS provided an important milestone for the GPELF and laid the groundwork for countries to scale down MDA, implementation unit by implementation unit, in a standardized fashion and to conduct post-MDA surveillance through the TAS 2 and TAS 3 surveys. It also provided a useful metric for programme success that could be captured in the GPELF summary published each year by the WHO in the Weekly Epidemiological Record.^14^ Based on its robust design, TAS has also provided a valuable survey platform to support the data collection for other public health priorities as well.^15,16^

## Refining the guidance—the need for research persists

As the LF programme has matured and countries have made substantial progress toward elimination, new challenges have been identified that require refining or adapting the existing guidance for countries. The research community has worked effectively with the GPELF and programme managers to address these challenges, in some cases generating improved strategies and in others highlighting important areas where research is still needed.

### Mapping

The initial guidance on LF mapping was biased towards starting MDA in any area where LF was thought to be present, so that all at-risk populations would be reached. Importantly, while the unit for mapping (and subsequently for programme implementation) could be defined as appropriate for the national programme, most often it was an administrative district. Programme managers were encouraged to use existing historical data and key informant interviews to determine where transmission was present and MDA needed. In areas of uncertain transmission status, the initial programme managers’ guidelines^9^ had recommended testing a sample of 250 school children (age not specified) in each implementation unit for filarial antigen. This guidance was used in some countries, but in most settings the approach to mapping LF that was adopted employed convenience sampling of adults in two communities in each district, selected based on either the presence of clinical disease or having environmental conditions conducive to transmission. This strategy served the global LF programme well as a low-cost strategy for scaling up MDA, particularly in settings where the index of suspicion that LF was present was high. Unfortunately the strategy was less useful where programme managers lacked LF-specific information, leaving a number of countries with districts whose mapping results were not sufficiently convincing to make decisions about whether or not to begin MDA. In the absence of clear guidance, an expert group was convened by the WHO to review standard approaches to LF mapping and to develop an alternative strategy that provided a more robust assessment of LF status. A protocol, adapted from the TAS, was developed based on the serologic testing of school-age children (9–14 y of age) for filarial antigen or antifilarial antibody. The presence of positive results among this age group of children provides stronger evidence of recent transmission than when adults are tested. This new ‘confirmatory mapping survey’ was designed to provide programme managers with yes/no decisions with respect to the need for MDA. After the survey was piloted in Ethiopia and Tanzania, use of the methodology was reviewed and endorsed by the WHO's NTD STAG and then adopted by partners and endemic countries.^17,18^ Adoption of the confirmatory mapping protocol (also referred to as the mini-TAS) has translated into significant resource savings where the need for MDA for LF was averted based on the findings with this new protocol.

### Monitoring programme coverage

When NTD programmes were first launched as disease-specific efforts and later integrated across platforms, the WHO and the endemic countries were focused on scaling up interventions to reach all at-risk populations. For the LF programme, the focus on a goal of elimination had been established from the outset and required that high levels of participation in MDA be achieved. Although the 2000 guidelines did not set a target goal for coverage, assessment of coverage was noted to be a critical programme element. Similarly the 2005 guidelines did not establish a specific coverage target but did provide coverage definitions as well as suggested coverage survey designs. The 2011 WHO guidelines were the first to establish a formal coverage target, and although coverage surveys were recommended, these were not often used by programmes.^13^ Empiric evidence to support specific coverage targets is still largely lacking, and the importance of ‘systematic noncompliance’, although recognized as a potential obstacle to achieving success,^19^ still has not been well established.

In an effort to provide more standardized guidance to programme monitoring, the WHO's M&E Working Group focused on the development of tools to improve data quality, standardize coverage evaluation surveys and develop monitoring tools to facilitate supervision during MDA. From a multicountry study comparing the feasibility of three commonly used survey methodologies for conducting coverage surveys, a single survey method was chosen to ensure that surveys were standardized. Similar efforts to pilot ‘data quality assurance’ evaluations and the ‘supervisor's coverage tool’ culminated in the release of a package of monitoring tools by the WHO, all of which contribute to the generation of more robust data and more effective MDAs.^20^

### TAS

The appeal of the TAS derives from the facts that it facilitates decision making, has proven feasible to implement, is standardized and incorporates a statistically rigorous design. Since its integration into the national programmes in 2011, the TAS has seen wide-scale implementation across the GPELF; however, evidence suggests that there are some settings in which the TAS might not be sufficient for evaluating interruption of transmission because of the heterogeneity of transmission at the subdistrict level. In such settings, undetected transmission may lead to a ‘passing’ TAS 1 result, despite focalized ongoing transmission, and potentially set the stage for failures of TAS 2 or TAS 3.^21,22^ Recognition of this problem has stimulated two complementary research efforts: analysing the programmatic factors that lead to TAS failure and developing new strategies to improve delivery of MDA and studying how to strengthen the TAS by modifying the sampling design or the choice of diagnostic tool. A related issue is how to interpret and respond to positive individual test results in a TAS 2 or TAS 3 that ‘passes’. While a passing TAS result suggests that any persistent, low-level transmission is so minimal that it is unlikely to present a risk of recrudescence, antigen-positive children in TAS 2 and TAS 3 represent evidence of ongoing infection in the community and are a definite cause of concern for NTD programme managers.

The introduction of triple-drug therapy with ivermectin, diethylcarbamazine and albendazole (IDA) for LF represents an innovation with the potential to facilitate elimination of LF because of the long-lasting impact that the combination of all three drugs has on circulating microfilariae.^23^ Modelling studies suggest that IDA may reduce the number of MDA rounds required to stop MDA, accelerating the drive toward LF elimination.^24^ Current guidance recommends monitoring in sentinel and spot-check sites after only two rounds of IDA.^25^ However, because the TAS uses antigen detection rather than circulating microfilariae as its indicator of infection, and since antigen clearance lags behind microfilarial clearance, antigenemia in children might not be the ideal indicator to determine IDA effectiveness. A modification to the TAS design will be needed in some settings where IDA is introduced, and this too is an area under active investigation.

### Surveillance

The current recommendation for programme surveillance for the GPELF is based on repeated use of the TAS after stopping MDA. The recommendation was made largely because there was very limited evidence to support other approaches to surveillance.^14^ TASs are not powered to detect changes in infection prevalence over time and specific follow-up strategies for antigen-positive children have not yet been established, consequently the effectiveness of TAS 2 and TAS 3 as surveillance tools is limited. Research efforts to strengthen the TAS include work to utilize the spatial data from TAS to inform, ‘adaptively’, the design of TAS 2 and TAS 3. Such an approach, in principle, would establish a stronger foundation for identifying ‘hotspots’ and targeting surveillance efforts more effectively.

Additional research is very much needed to determine how improved surveillance can support the documentation of interruption of transmission and create a pathway for programmes to satisfy the WHO criteria for ‘verification’ of LF elimination at the national level.^3^ Efforts to strengthen surveillance would, moreover, be augmented by the development and deployment of new diagnostic tools, including both serologic tools and a clearer understanding of whether there is a role for using xenomonitoring (i.e. detecting LF parasites in the vector mosquitoes) at the programmatic level. Surveillance is a cross-cutting issue that has been addressed as a priority by the WHO's new Diagnostic and Technical Advisory Group, and an updated set of target product profiles to guide development of new diagnostic tools has now been developed.^26^

Over the years the GPELF has embraced the concept that an active and collaborative research programme is a necessary component of an effective M&E framework to support a disease elimination programme. This commitment to ongoing research adds to the confidence that the gains already achieved by the GPELF will be sustained in the future and that elimination of LF transmission, at the global level, is an achievable goal.

## Data Availability

None.
